# E-cadherin maintains the undifferentiated state of mouse spermatogonial progenitor cells via β-catenin

**DOI:** 10.1186/s13578-022-00880-w

**Published:** 2022-09-01

**Authors:** Weixiang Song, Danchen Zhang, Jiaqi Mi, Wenfei Du, Yang Yang, Rong Chen, Cong Tian, Xiaodong Zhao, Kang Zou

**Affiliations:** 1 Germline Stem Cells and Microenvironment Lab, College of Animal Science and Technology, Nanjing Agricultural University, Nanjing, 210095 China; 2 Stem Cell Research and Translation Center, Nanjing Agricultural University, Nanjing, 210095 China; 3 Department of Cancer Biology, Cancer Center and Beckman Research Institute, City of Hope, 91010 Duarte, USA; 4 Key Laboratory of Systems Biomedicine (Ministry of Education), Shanghai Center for Systems Bio-medicine, Shanghai Jiao Tong University, Shanghai, 200240 China

**Keywords:** E-cadherin, ß-catenin, SPC differentiation, Transcription regulation

## Abstract

**Background:**

Cadherins play a pivotal role in facilitating intercellular interactions between spermatogonial progenitor cells (SPCs) and their surrounding microenvironment. Specifically, E-cadherin serves as a cellular marker of SPCs in many species. Depletion of *E-cadherin* in mouse SPCs showed no obvious effect on SPCs homing and spermatogenesis.

**Results:**

Here, we investigated the regulatory role of E-cadherin in regulating SPCs fate. Specific deletion of *E-cadherin* in germ cells was shown to promote SPCs differentiation, evidencing by reduced PLZF^+^ population and increased *c-Kit*^+^ population in mouse testes. *E-cadherin* loss down-regulated the expression level of *β-catenin*, leading to the reduced β-catenin in nuclear localization for transcriptional activity. Remarkably, increasing expression level of Cadherin-22 (CDH22) appeared specifically after *E-cadherin* deletion, indicating CDH22 played a synergistic effect with E-cadherin in SPCs. By searching for the binding partners of β-catenin, Lymphoid enhancer-binding factor 1 (LEF1), T-cell factor (TCF3), histone deacetylase 4 (HDAC4) and signal transducer and activator 3 (STAT3) were identified as suppressors of SPCs differentiation by regulating acetylation of differentiation genes with PLZF.

**Conclusions:**

Two surface markers of SPCs, E-cadherin and Cadherin-22, synergically maintain the undifferentiation of SPCs via the pivotal intermediate molecule β-catenin. LEF1, TCF3, STAT3 and HDAC4 were identified as co-regulatory factors of β-catenin in regulation of SPC fate. These observations revealed a novel regulatory pattern of cadherins on SPCs fate.

**Supplementary Information:**

The online version contains supplementary material available at 10.1186/s13578-022-00880-w.

## Introduction

Spermatogonial stem cells (SSCs) are the foundation of spermatogenesis, which are able to differentiate into functional sperms via multiple steps in testis. In their differentiation hierarchies, A_s_, A_pr_ and A_al_ spermatogonia are referred to as the undifferentiated populations, and are nominated as spermatogonial progenitor cells (SPCs) [1]. A transcription suppressor promyelocytic leukemia zinc finger (PLZF) is identified as a specific SPC marker [2, 3], and further confirmed to be an essential factor for SPCs maintenance [4], through binding to and inhibiting many differentiation associated genes such as *c-Kit* [5], *Sall4* [1] and *Redd1* [6]. Based on these findings, our recent study successfully identified additional target genes of PLZF that are associated with SPC differentiation, including *Stra8*, *Sohlh2* and *Dmrt1* [7].

SPCs fate is regulated by their microenvironment through interacting with neighboring cells. And many membrane or transmembrane proteins, such as cadherins, integrins, claudins, are intimately associated with intra- and intercellular functions such as adhesion, binding, recognition and signal transduction [8, 9]. Among them, E-cadherin acts as an important molecule involved in structural and signaling-related functions in epithelial cells, SSCs included [8], and has been characterized as a SPC marker [10]. As a transmembrane molecule, E-cadherin is involved in binding of SPCs to the niche and regulating SPC’s fate [11, 12]. Our recent study revealed that E-cadherin on SPCs could interact with ITGB1 on Sertoli cells [13]. However, evidence from conditional knockout of E-cadherin in SSCs showing no impact on SSCs homing after transplantation cannot lead to the conclusion that E-cadherin is ineffective for SPCs, due to the fact that SPCs are enriched with other cadherins which could compensate for E-cadherin loss [14]. Nevertheless, germline specific deletion of E-cadherin at embryonic stage leads to germ cell loss caused by apoptosis, indicating that a pivotal role of E-cadherin in gonad development [15]. Here, we were wondering if E-cadherin contributed to the regulation of SPC’s fate, especially as a binding partner of β-catenin, another pivotal player in the proliferation of SPCs [16]. Interestingly, evidence from another group suggested that β-catenin was more likely to promote differentiation of SPCs [17] by using a β-catenin overexpression model. In their case, the role of ß-catenin was explored in a more Wnt signaling regulatory-related way. More observations showed that hyperactivation of Wnt/β-catenin signaling in gonocyte [18], spermatogonial cell line [19] or in vivo [20] resulted in reduced cell proliferation and viability, indicating an enhanced exhaustion of SSCs pool. As a multiple-role molecule, β-catenin was proven to possess structural and signaling-associated functions, via interacting with many signaling pathways (Wnt, JAK-STAT, etc.) and transcriptional factors (TCF family, HDAC family, etc.). These complicated interactions shadow our understanding of the molecular mechanism of β-catenin, especially in the regulation of stem cell fate [21]. Thus, further study is required to unveil the comprehensive mechanism of β-catenin in SPCs.

β-catenin functions as a pivotal intermediate molecule in Wnt signaling pathway [22, 23]. However, its binding to cadherins as a structural component can also play a role as co-transcription factor in a dynamic pattern [9]. Notably, β-catenin relies on a mediator to convey its regulatory effect on the expression of target genes due to a lack of DNA binding domain [23]. In addition to the well-known TCF family, β-catenin could cooperate with HDAC family members as well. Some HDACs are found to be involved in functions of germline, for instance the expression levels of *Hdac2*, *Hdac6*, and *Sirt1* increased, while *Hdac8*, *Hdac9* and *Sirt4* decreased, during SSCs differentiation or aging [24]. Moreover, a study reported that HDAC4 bound to PLZF to enhance the repression of differentiation [25]. Thus, it might be interesting to look into the interplay between β-catenin and HDAC in SPCs.

Here, the role of E-cadherin in SPCs was studied using conditional knockout mice and in vitro cell culture models. The dynamic role of β-catenin in regulating SPCs fate was identified, and the interaction regarding downstream genes or cooperators was further analyzed. Briefly, E-cadherin was identified as an essential transmembrane molecule to maintain undifferentiated state of SPCs, and CDH22 played a similar role as E-cadherin and might compensate for E-cadherin loss. Deletion of E-cadherin disturbed the dynamic balance of β-catenin in structural maintenance, protein degradation and nuclear localization, leading to a reduced β-catenin expression and promoted SPCs differentiation. Moreover, the interaction among β-catenin, PLZF and HDAC4 was discussed in SPCs. These observations indicated a new regulatory pattern of SPCs differentiation.

## Results

### E-cadherin and β-catenin were co-expressed in undifferentiated spermatogonia

**Fig. 1:**
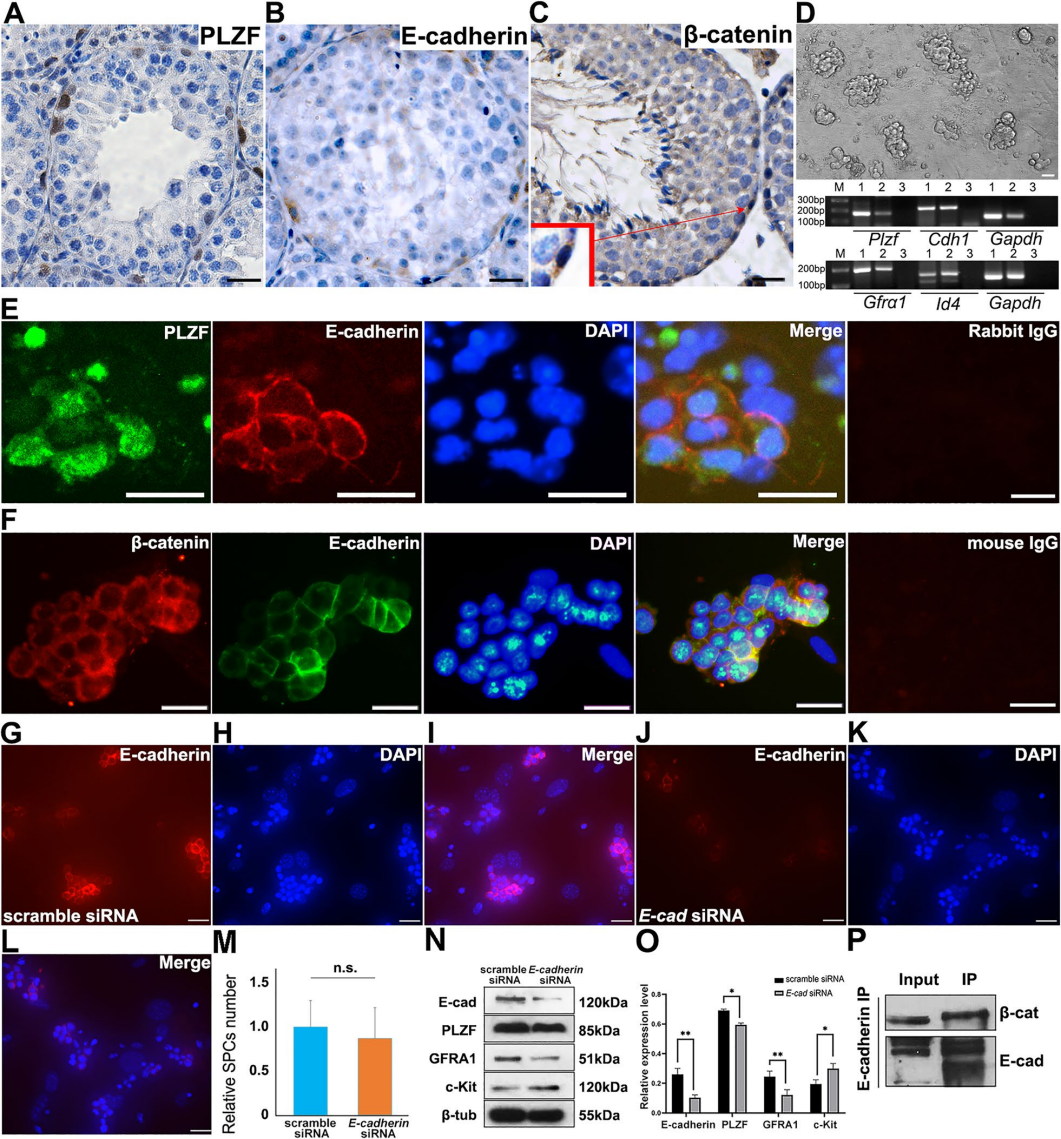
Expression of E-cadherin and SPC markers in mouse testis and isolated SPCs. The expression of PLZF (**A**), E-cadherin (**B**) and β-catenin (**C**) was detected in the testis of 42-day old mouse using IHC. The morphology of purified SPCs on MEF feeder layer was exhibited, and expression of SPCs markers was determined using RT-PCR (1.testis, 2.SPCs, 3.H_2_O) (**D**). Co-IF staining was employed to detect the expression of PLZF and E-cadherin (**E** green: PLZF, red: E-cadherin, blue: DAPI), or β-catenin and E-cadherin (**F** red β-catenin, green E-cadherin, blue DAPI) in purified SPCs. Expression of E-cadherin was detected in SPCs transfected with scrambled (**G** E-cadherin, H. DAPI, I. Merge) or E-cadherin siRNA (**J** E-cadherin, **K** DAPI, **L** Merge) was exhibited 72 h post transfection using IF staining. The number of SPCs was statistically calculated (**M**) (10 views of ×200 were randomly selected). The expression of E-cadherin, PLZF, GFRA1, c-Kit and β-tubulin was determined in scrambled or E-cadherin siRNA transfected SPCs using Western blot, n=3 (**N**), and was statistically calculated (**O**). The interaction of E-cadherin and β-catenin in SPCs was detected using co-IP (**P**). Scale bar = 20 μm, data represents mean ± standard deviation (SD), *p<0.05, **p<0.01

A subpopulation of undifferentiated spermatogonia, SPCs, was identified as PLZF^+^ cells residing in the first layer in seminiferous tubules and found to regulate SPCs fate (Fig. 1A). Since the expression of E-cadherin and β-catenin could be detected in the same population (Fig. 1B and C), we postulated that both molecules might be involved in modulating SPCs fate. To study the role of E-cadherin, SPCs were purified using THY1.2^+^ MACS from neonatal mouse testis, and grape-like clones were observed after 2 passages on MEF feeder layers (Fig. 1D), which were able to be stably maintained in vitro for more than 30 passages [26]. The expression of SPC markers, including *Plzf*, *Cdh1*, *Gfra1* and *Id4*, was examined to characterize their identities using RT-PCR (Fig. 1D). Moreover, IF staining against PLZF, E-cadherin, β-catenin, Axin2 and ZO-2 further confirmed that SPCs were notably enriched (Additional file 1: Fig.S1 A–E). Subsequently, co-IF staining demonstrated an overlap of E-cadherin^+^ and PLZF^+^/β-catenin^+^ populations (Fig. 1E and F). In all, these observations confirmed a co-expression pattern of E-cadherin and β-catenin both in vivo and in vitro, demonstrating that E-cadherin in combination of canonical Wnt signaling pathway might play a role in SPCs.

### Differentiation markers up-regulated after disturbing E-cadherin expression in SPCs

To better understand the role of E-cadherin, RNAi was employed to disturb the expression of *E-cadherin* in SPCs. IF staining revealed the efficient decrease of *E-cadherin* expression in primary SPCs (Fig. 1J–L), compared to scramble siRNA group (Fig. 1G–I). Although neither obvious morphological change (data not shown) nor difference in the number of SPCs was observed 72 h post-transfection (Fig. 1M), the expression of SPC markers was altered (Fig. 1N, O). Reduced PLZF and GFRA1 along with the increased differentiation marker c-Kit suggested that the disturbance of *E-cadherin* might jeopardize the undifferentiated state of SPCs under in vitro culture. The binding of E-cadherin to β-catenin was further confirmed in SPCs by co-IP (Fig. 1P). Though *E-cadherin* knockout maintained the capacities of SPC homing and spermatogenesis [14], our observations revealed a possible influence of E-cadherin on SPCs fate. Therefore, a better understanding of the regulatory mechanism of E-cadherin in SPCs could be greatly valued, especially its interaction with β-catenin.

### Conditional knockout of *E-cadherin* in germline promoted differentiation at protein levels

**Fig. 2:**
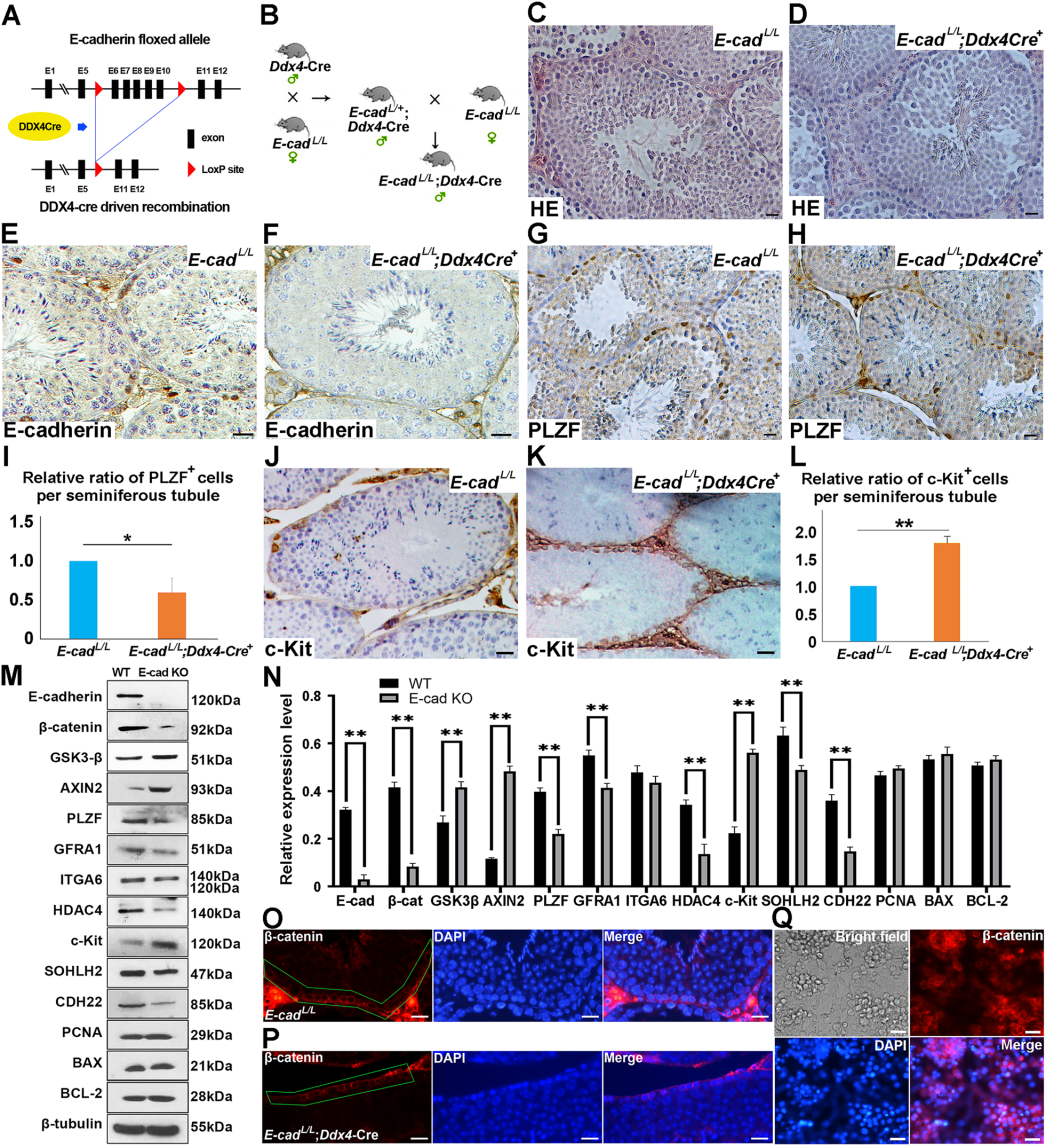
Expression of E-cadherin and SPC markers in germline-specific E-cadherin knockout mice. A schematic illustration of conditional knockout of *E-cadherin* driven by *Ddx4-Cre* in germ cells (**A**). The goal mice with E-cadherin germline specific knockout were generated by mating *E-cadherin* floxed and *Ddx4*-Cre mice (**B**). The histology of testes from 90-day old *E-cadherin*^L/L^ (**C**) and *E-cadherin*^L/L^;*Ddx4*-Cre^+^ mice (**D**) was exhibited. The expression of E-cadherin, PLZF and c-Kit in testes from 90-day old *E-cadherin*^L/L^ (**E** E-cadherin, **G** PLZF, **J** c-Kit) and *E-cadherin*^L/L^;*Ddx4*-Cre^+^ (**F** E-cadherin, H. PLZF, **K** c-Kit) were determined using IHC. The relative ratio of PLZF^+^ (**I**) and c-Kit^+^ (**L**) cells per seminiferous tubule in testes from both genotypes was statistically analyzed (for each genotype, 15 tubules from 3 testes were counted). The expression levels of E-cadherin, β-catenin, GSK3-β, AXIN2, PLZF, GFRA1, ITGA6, HDAC4, c-Kit, SOHLH2, CDH22, PCNA, BAX, BCL-2 and β-tubulin were determined in testes from both genotypes at 90-day using Western blot, n = 3 (**M**), and was statistically analyzed (**N**). Expression of β-catenin in testes from 90-day old *E-cadherin*^L/L^ (**O**) and *E-cadherin*^L/L^;*Ddx4*-Cre^+^ mice (**P**) and newly isolated SPCs (**Q**) was determined using IF. Scale bar = 20 μm, data represent as mean ± SD *p < 0.05; **p < 0.01

To further characterize the effect of *E-cadherin* deficiency on SPCs fate, LoxP-Cre system was employed to conditionally knockout *E-cadherin* in mouse SPCs (Fig. 2A). Germline-specific *E-cadherin* knockout mice (*E-cadherin*^L/L^;*Ddx4-Cre*^+^) were generated by mating *E-cadherin* floxed females with *E-cadherin*^*L/*+^*;Ddx4-Cre*^+^ males (Fig. 2B). Testes from 3-month *E-cadherin*^L/L^;*Ddx4-Cre*^+^ males were harvested for histological analysis, and germ cells at different differentiation stages could be easily distinguished (Fig. 2C–F), indicating that *E-cadherin* deficiency neither affected seminiferous tubule structure nor disturbed spermatogenesis. However, when evaluating the expression of undifferentiated spermatogonia marker PLZF using IHC, we noticed that the number of PLZF^+^ cells in *E-cadherin*^L/L^;*Ddx4*-*Cre*^+^ testis remarkably decreased compared to control group (Fig. 2G–I), and the number of differentiating population represented by c-Kit staining intensively increased in *E-cadherin* deficient tubules (Fig. 2J–L). These observations demonstrated that E-cadherin might inhibit SPCs differentiation. To obtain a better understanding, testes from *E-cadherin*^L/L^ and *E-cadherin*^L/L^;*Ddx4*-*Cre*^+^ littermates were collected for evaluating the expression of self-renewal and differentiation markers using Western blot. Consistently, *E-cadherin* deficient testes expressed decreasing level of SPC markers such as GFRA1, PLZF and ITGA6, and increasing expression level of differentiation marker c-Kit compared to control group, respectively (Fig. 2M and N). Noteworthy, expression of AXIN2 and GSK3-β was up-regulated (Fig. 2M and N), suggesting that *E-cadherin* deficiency possibly enhanced the activation of β-catenin degradation complex. Meanwhile, the expression of PCNA, BAX and BCL-2 was not affected in *E-cadherin* deficient group (Fig. 2M and N), implying that *E-cadherin* loss promoted differentiation, but not proliferation or apoptosis, in testis.

Subsequently, we explored the impact of *E-cadherin* deficiency on β-catenin’s translocation into nucleus. IF staining of β-catenin in *E-cadherin*^L/L^ and *E-cadherin*^L/L^;*Ddx4*-*Cre*^+^ testes revealed limited nuclear distribution of β-catenin, while no remarkable difference observed between wild type and *E-cadherin* deficient SPCs (Fig. 2O and P). Likewise, strong β-catenin signal was restricted to cytoplasm of purified SPCs (Fig. 2Q), we postulated that β-catenin was mainly distributed in cytoplasm in both genotypes. Collectively, E-cadherin could play an important role in SPCs differentiation by modulating both differentiation and SPC marker expression.

### The impact of *E-cadherin* deficiency on the fates of β-catenin in SPCs

**Fig. 3:**
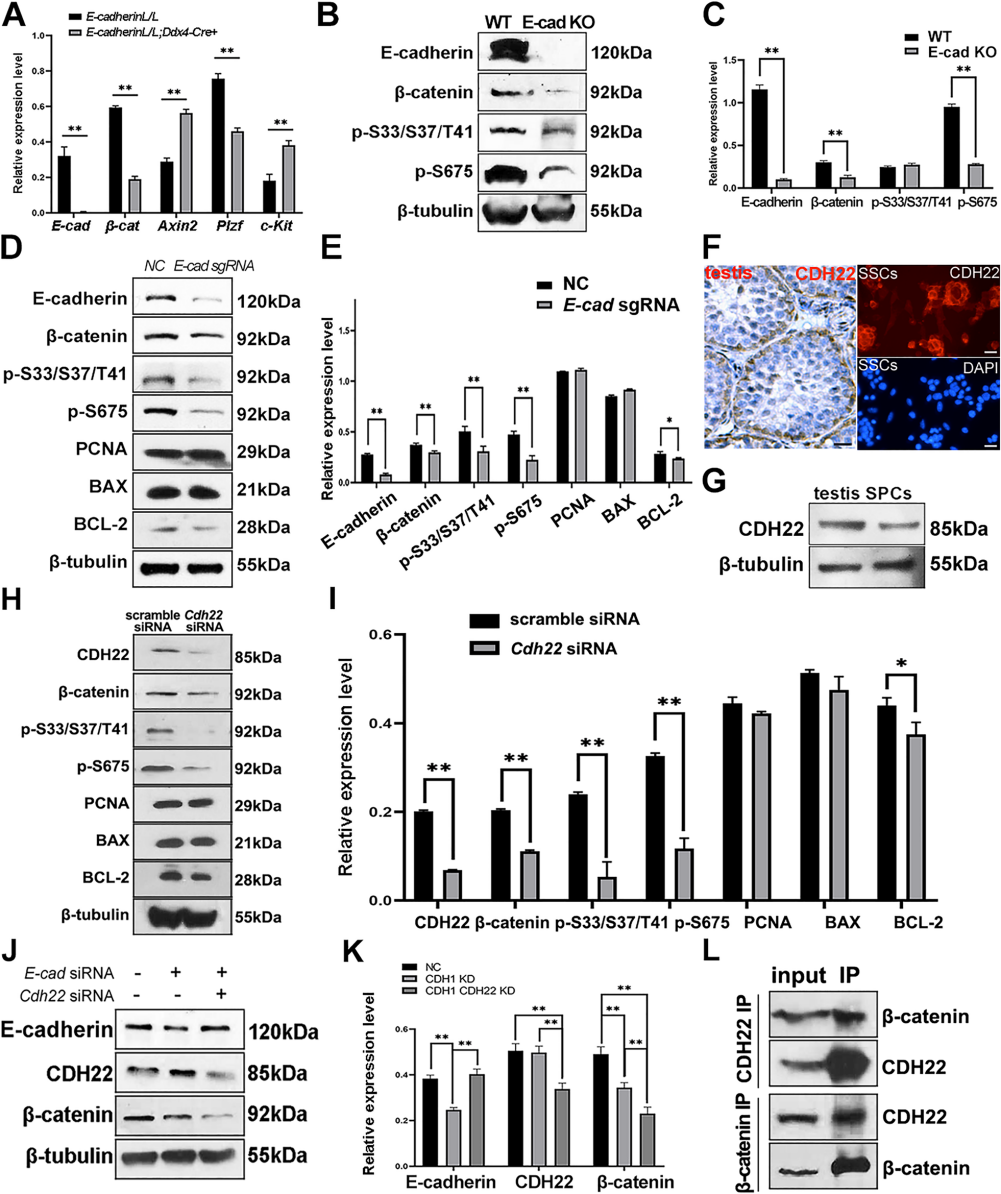
The expression of β-catenin was regulated by *E-cadherin* knockout in SPCs. Expression levels of *E-cadherin*, *β-catenin*, *Axin2*, *Plzf* and *c-Kit* mRNA were detected in SPCs from *E-cadherin*^L/L^ and *E-cadherin*^L/L^;*Ddx4*-Cre^+^ mice using real time-qPCR (**A**). The expression levels of E-cadherin, β-catenin, and β-catenin phosphates (S33/S37/T41 and S675) were detected in SPCs from both genotypes (**B**) using Western blot, and were statistically analyzed (**C**). The expression levels of E-cadherin, β-catenin, and β-catenin phosphates (S33/S37/T41 and S675), PCNA, BAX, BCLL-2 and β-tubulin in *E-cadherin* deleted SPCs mediated CRISPR/Cas9 were detected using Western blot, n=3 (**D**), and were statistically analyzed (**E**). CDH22 was detected in 20-day mouse testes (left) and freshly isolated SPCs from 5-day mice (right, top: CDH22, down: DAPI) (**F**). A single band of CDH22 was detected in mouse testis and SPCs (**G**). Phosphorylation at S33/S37/T41 and S675 of β-catenin, and expression levels of CDH22, β-catenin, PCNA, BAX, BCL-2 and β-tubulin were detected in SPCs transfected with scramble or *E-cadherin* siRNA (**H**), and were statistically analyzed (**I**). Expression of E-cadherin, CDH22 and β-catenin in SPCs transfected with scramble, *E-cadherin* siRNA, or *E-cadherin* siRNA plus *Cdh22* siRNA was evaluated with Western blot, n=3 (**J**), and were further statistically analyzed (**K**). The binding of CDH22 and β-catenin was verified with co-IP (**L**). Scale bar = 20 μm, data represent as mean ± SD, *p<0.05, **p<0.01

A decreased β-catenin expression in *E-cadherin* knockout SPCs compared with WT controls might be detected because of two possibilities: 1. *E-cadherin* knockout led to reduced expression of β-catenin; 2. *E-cadherin* knockout enhanced the degradation of β-catenin. To test these hypotheses, we first compared the expression of *β-catenin* at mRNA level in SPCs from both genotypes, and noticed an attenuated *β-catenin* expression in *E-cadherin* knockout SPCs (Fig. 3A). Subsequently, different phosphorylated forms of β-catenin were determined. A β-catenin antibody targeting phosphorylation at Ser33/Ser37/Tyr41 was used to examine the degradation of β-catenin, and the declined phosphorylation implied that *E-cadherin* knockout reduced the degradation of β-catenin in SPCs (Fig. 3B and C). Interestingly, phosphorylation at Ser675 representing a transcriptional active form of β-catenin, was declined in *E-cadherin* knockout SPCs as well (Fig. 3B and C), indicating that *E-cadherin* deficiency down-regulated the transcriptional activity of β-catenin. Furthermore, a sustained effect on β-catenin expression and phosphorylation was observed when *E-cadherin* got deleted in cultured SPCs with CRISPR/Cas9 (Fig. 3D, E). The expression of PCNA and BAX was not changed, while expression of anti-apoptosis protein BCL-2 was down-regulated. Thus, we proposed that E-cadherin might play a role in anti-apoptosis, since the ratio of BCL-2/BAX reduced after *E-cadherin* loss. Considering that *E-cadherin* deletion increased Axin2 and GSK-3β expression in testes (Fig. 2M, N), we concluded that *E-cadherin* knockout down-regulated *β-catenin* expression, resulting in a reduced transcriptional activity of β-catenin.

### CDH22 co-regulates β-catenin with E-cadherin in SPCs

In addition to E-cadherin, we were also interested in other types of cadherins expressed in SPCs, particularly CDH22, a key signal molecule regarding SPCs fate [27]. In rats, *Cdh22* encodes two splicing proteins. The shorter one lacking catenin binding domain is associated with SSCs self-renewal through interacting with JAK-STAT and PI3K-AKT signaling pathways, while the longer one contains catenin binding domain [27]. Notably, *Cdh22* in mouse ovary only encodes the latter one, which interacts with β-catenin to regulate female germline stem cells (FGSCs) self-renewal [28]. Here, we wondered whether CDH22 could compensate for *E-cadherin* loss in SPCs. As shown in Fig. 3F, CDH22 was detected in SPCs residing in basal membrane and freshly isolated SPCs. Western blot results revealed that only one band was detected in mouse SPCs (Fig. 3G), which was consistent with that of mouse FGSCs. Subsequently, we disturbed *Cdh22* expression in SPCs and confirmed the reduced phosphorylation levels of S33/S37/T41 and S675 of β-catenin (Fig. 3H, I), and decreased expression of anti-apoptotic protein BCL-2 (Fig. 3H, I), indicating that CDH22 was positively correlated with transcription activity of β-catenin and anti-apoptosis capacity in SPCs, similar to E-cadherin. Also, simultaneous transfection of *Cdh1* and *Cdh22* siRNA into SPCs aggravated the decline of β-catenin expression (Fig. 3J and K), suggesting that CDH22 might regulate β-catenin expression in SPCs along with E-cadherin in a synergistic manner. More importantly, the binding of CDH22 and β-catenin was confirmed using co-IP (Fig. 3L), indicating a direct interaction between CDH22 and β-catenin in SPCs. Based on these observations, we postulated that β-catenin could be a critical intermediate molecule interacting with CDH1 and CDH22 to regulate SPCs fate.

### Identification of ß-catenin co-regulatory factors in SPCs

**Fig. 4:**
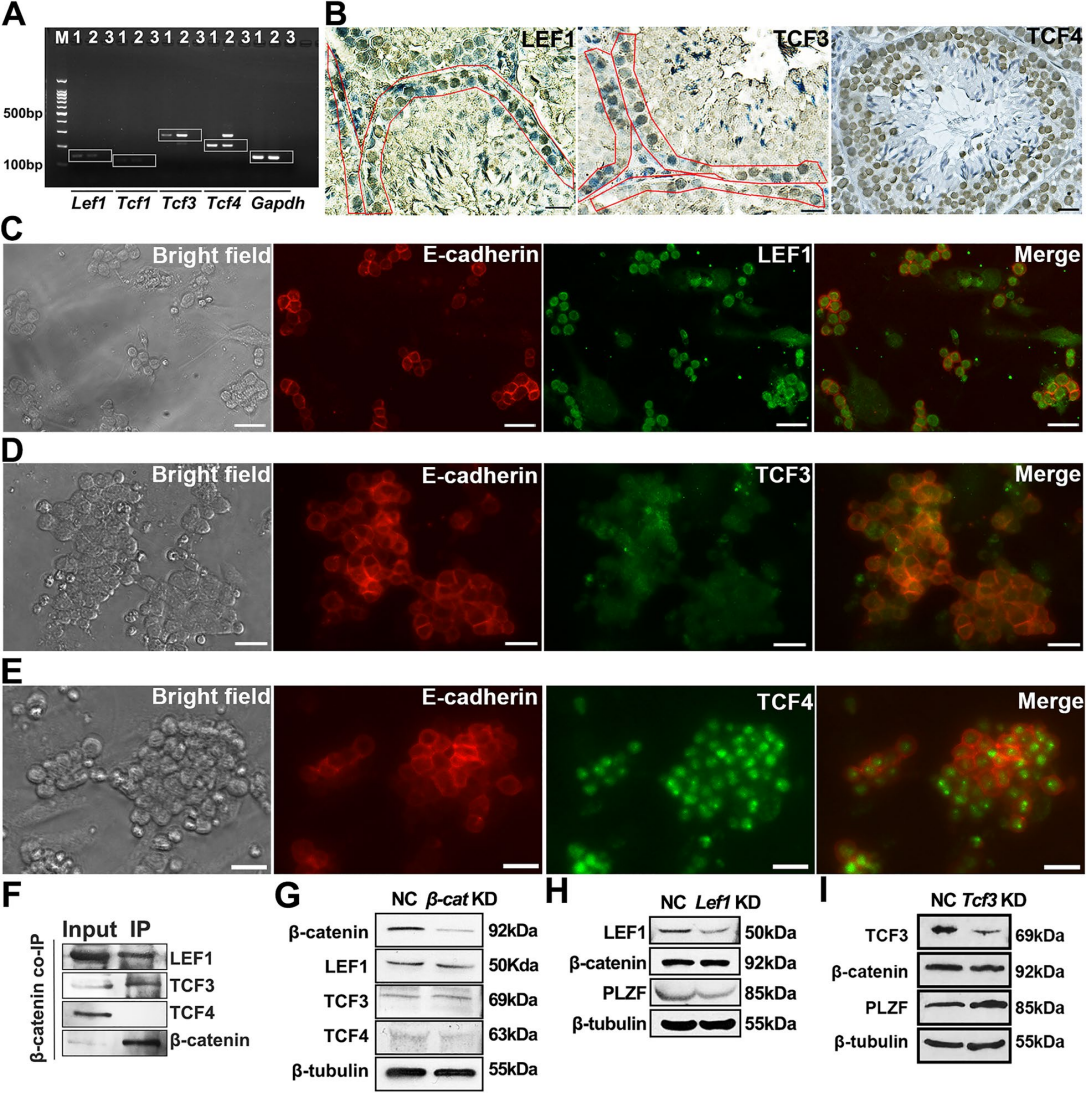
The binding between β-catenin and TCF family in SPCs. Expression of *Tcf* family was determined in SPCs using RT-PCR (M: marker; 1: testis; 2: SPCs; 3: negative control) (**A**). The expression of LEF1, TCF3 and TCF4 was detected in 42-day testis using IHC (SPCs populations residing in basal membrane were enclosed in red frames) (**B**). Co-IF staining demonstrated the colocalization of E-cadherin/LEF1 (**C**), E-cadherin/TCF3 (**D**) and E-cadherin/TCF4 (**E**) in purified SPCs. The binding between β-catenin and LEF1/TCF3/TCF4 was examined with co-IP (**F**). The expression of LEF1, TCF3 and TCF4 was determined in scrambled or β-catenin siRNA treated SPCs using Western blot (**G**). The expression of β-catenin and PLZF was detected in scrambled or *Lef1/Tcf3* siRNA treated SPCs, respectively (**H**, **I**). Scale bar = 20 μm

Due to lack of DNA binding domain, β-catenin needs to bind to TCF family including LEF1, TCF1, TCF3 and TCF4 in mouse and human to regulate target gene expression [21]. Using RT-PCR, *Lef1*, *Tcf3* and *Tcf4* mRNA were detected in SPCs (Fig. 4A). Interestingly, LEF1 and TCF3 were restricted to SPCs residing in the basal membrane, while TCF4 was broadly distributed in undifferentiated spermatogonia, differentiating spermatogonia and mature spermatocytes (Fig. 4B). IF staining confirmed the expression of LEF1, TCF3 and TCF4 in purified SPCs (Fig. 4C–E), and co-IP assays demonstrated the binding of β-catenin to LEF1 and TCF3, but not TCF4 in SPCs (Fig. 4F). As shown in Fig. 4G, *β-catenin* knockdown in SPCs showed no impact on the expression of LEF1, TCF3 and TCF4. On the other hand, though decreased expression of LEF1 led to no significant change in β-catenin expression, a down-regulation of PLZF was observed (Fig. 4H). Considering β-catenin combined with LEF1 regulates *Plzf* expression in innate memory-like CD8 thymocytes [29], a similar regulatory pattern might exist in SPCs to maintain the undifferentiated state. Also, we hypothesized that β-catenin displaced the suppressor TCF3 from self-renewal associated genes (such as *Plzf*). Conversely, knockdown of *Tcf3* caused up-regulation of PLZF (Fig. 4I), suggesting opposite roles of LEF1 and TCF3 in regulating SPCs fate by cooperating with β-catenin. Our current finding raises up a question that whether β-catenin displaces its suppressor TCF3 from binding to self-renewal associated genes (such as *Plzf*) to maintain SPCs undifferentiated state, which requires further investigation.

### Validation of co-regulatory role of HDAC4 with PLZF in SPCs

**Fig. 5:**
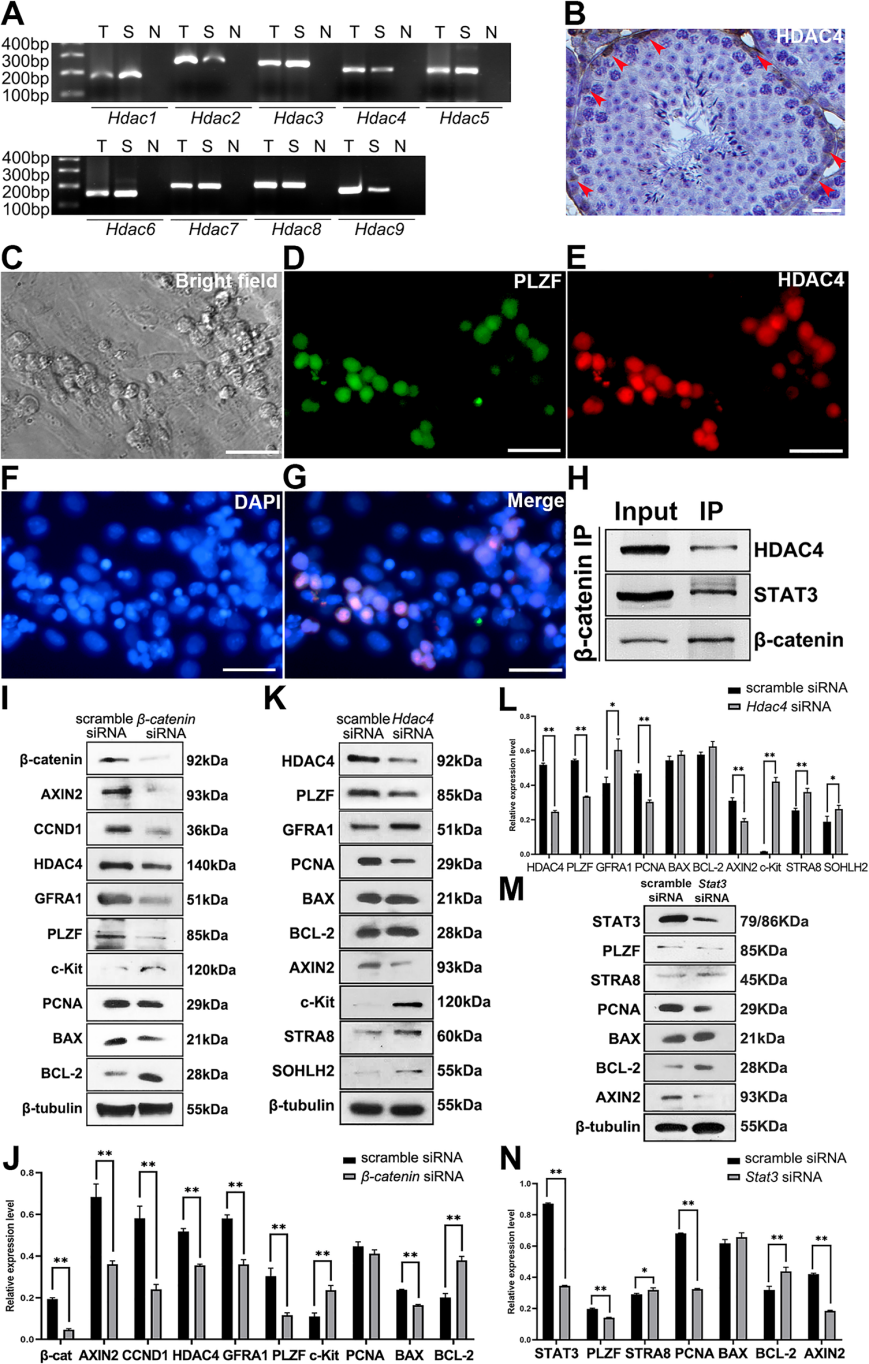
β-catenin regulated the expression of SPC markers and HDACs. The mRNA expression of *Hdac*1-9 was determined in SPCs using RT-PCR (T: testis; S: SPCs; N: negative control) (**A**). Expression of HDAC4 in testis was detected from 42-day old mouse using IHC (**B**). Co-IF staining demonstrated the expression and colocalization of PLZF and HDAC4 in purified SPCs (**C**–**G**). The binding of β-catenin to HDAC4 and STAT3 was examined using Co-IP (**H**). The expression levels of β-catenin, AXIN2, Cyclin D1, HDAC4, GFRA1, PLZF, c-Kit, PCNA, BAX, BCL-2 and β-tubulin were determined in SPCs treated with scrambled or *β-catenin* siRNA using Western blot, n=3 (**I**), and were statistically analyzed (**J**). Western blot was employed to detect the expression levels of HDAC4, PLZF, GFRA1, PCNA, BAX, BCL-2, AXIN2, c-Kit, STRA8, SOHLH2 and β-tubulin in scrambled or *Hdac4* siRNA transfected SPCs, n=3 (**K**), and the results were statistically analyzed (**L**). The expression levels of STAT3, PLZF, STRA8, PCNA, BAX, BCL-2, AXIN2 and β-tubulin were determined in SPCs transfected with scrambled or *Stat3* siRNA, n=3 (**M**), and were statistically analyzed (**N**). Scale bar = 20 μm, data represent as mean ± SD, *p<0.05,**p<0.01

In addition to TCF family, β-catenin is able to cooperate with other co-factors as well, such as SOX1, SOX2 and KLF4 [21]. Among them, we were specifically interested in HDAC family, known as pivotal partners in regulating gene expression [30], especially in germline [31]. Consequently, we wondered if HDAC was able to directly bind to β-catenin as a cooperator. Indeed, purified SPCs expressed *Hdac1-9* mRNA (Fig. 5A), and HDAC4 was predominately expressed in SPCs residing in the basal membrane of seminiferous tubules (Fig. 5B). In purified SPCs, HDAC4 signal was highly overlapped with PLZF in the nucleus (Fig. 5C–G) demonstrating the co-localization of HDAC4 and PLZF. Subsequently, co-IP assay revealed the binding of β-catenin and HDAC4 in SPCs, as well as STAT3 (Fig. 5H), another key transcription factor for SSCs self-renewal and differentiation via cooperation with the β-catenin/TCF4 complex [32]. To understand the interaction between β-catenin and HDAC4, *β-catenin* knockdown was performed in SPCs, resulting in a slightly increased expression of c-Kit and BCL-2, as well as decreased GFRA1, PLZF, Cyclin D1, HDAC4 and BAX (Fig. 5I and J), implying differentiation was enhanced, but proliferation and apoptosis were declined in SPCs. This observation is consistent with a previous study showing that hyper-proliferation is accompanied with enhanced apoptosis in Wnt hyper-active gonocytes [18]. Meanwhile, *RNAi* assay was employed to reveal the role of HDAC4 in SPCs, and the results showed that the growth condition of SPCs transfected with *Hdac4* siRNA was not remarkably altered during 48 h post transfection compared with control (data not shown), but the expression of PLZF was suppressed, and the expression of differentiation markers including c-Kit, STRA8 and SOHLH2, were up-regulated (Fig. 5K and L). Notably, *Hdac4* loss led to down-regulation of PCNA and AXIN2, without affecting apoptosis (Fig. 5K and L), suggesting that HDAC4 is more likely a regulator to maintain SPCs self-renewal, and is probably associated with canonical Wnt signal pathway. Collectively, these observations indicated a positive correlation between HDAC4 and β-catenin expression in SPCs, which might synergistically regulate SPCs differentiation, proliferation or apoptosis. Thus, we proposed that β-catenin combined with HDAC4 in SPCs to maintain the undifferentiation state and proliferation capacity.

Similarly, STAT3 might be involved in the regulation of differentiation and proliferation in SPCs, since disturbance of *Stat3* also led to decreasing PLZF and PCNA, and increasing STRA8 (Fig. 5M and N). Moreover, STAT3 in SPCs seems to maintain β-catenin activity, since *Stat3* loss caused decreased AXIN2 (Fig. 5M and N). The increased value of BCL-2/BAX implied that *Stat3* loss strengthened the anti-apoptosis capacity in SPCs, further confirming that the positive correlation of proliferation and apoptosis in SPCs. Overall, these observations suggested that HDAC4 and STAT3 could be potential collaborators of β-catenin that synergically regulated SPCs fate.

### HDAC4 directly repressed *c-Kit* expression through deacetylation in SPCs

**Fig. 6:**
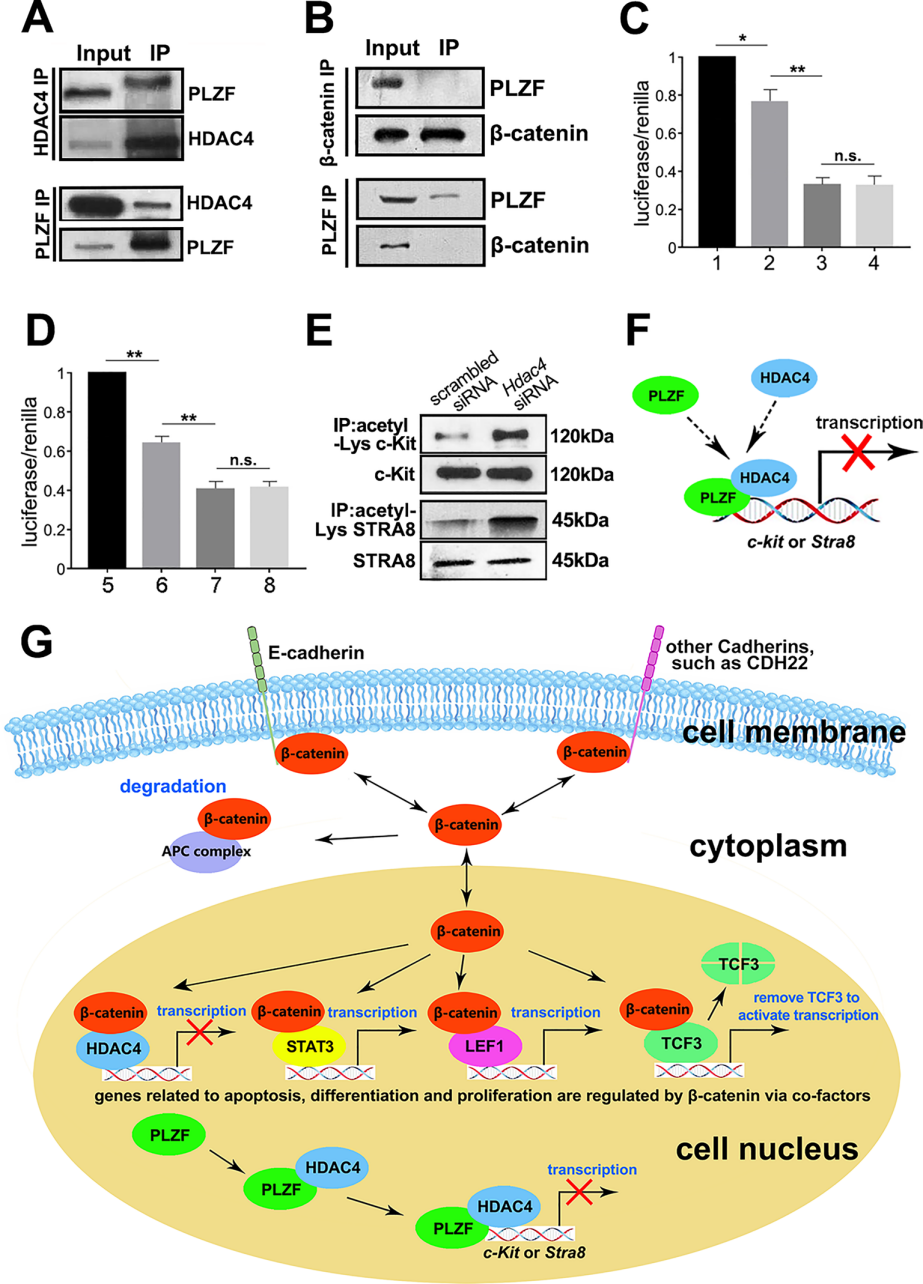
HDAC4 suppressed *c-Kit* expression via deacetylation. The binding of PLZF and HDAC4 (**A**), β-catenin and PLZF (**B**) were determined in SPCs using co-IP. The inhibitory effect of PLZF and HDAC4 on *c-Kit* (**C**) and *Stra8* (**D**) was evaluated in dual-luciferase assay (1. pGL-*c-Kit* + pcDNA3.1, 2. pGL-*c-Kit* + pcDNA3.1-*Plzf*, 3. pGL-*c-Kit* + pcDNA3.1-*Hdac4*, 4. pGL-*c-Kit* + pcDNA3.1-*Plzf* + pcDNA3.1-*Hdac4;* 5. pGL-*Stra8* + pcDNA3.1, 6. pGL-*Stra8* + pcDNA3.1-*Plzf*, 7. pGL-*Stra8* + pcDNA3.1-*Hdac4*, 8. pGL-*Stra8* + pcDNA3.1-*Plzf* + pcDNA3.1-*Hdac4*), n=3. Acetylation lysine IP coupled with Western blot was used to detect the impact of HDAC4 on c-Kit acetylation (**E**). A schematic illustration described the regulatory mechanism that HDAC4 could directly bind to *c-Kit* to suppress its expression via deacetylation (**F**). The regulatory pattern of cadherins on SPCs fate was schematically summarized (**G**). Data represent as mean ± SD *p < 0.05; **p < 0.01

Since HDAC family members could cooperate with transcription factors [25], we investigated whether HDAC4 bound to differentiation suppressor PLZF in SPCs. Co-IP assay confirmed the binding of HDAC4 to PLZF in SPCs (Fig. 6A), suggesting HDAC4 might be a co-suppressor of PLZF in the inhibition of SPCs differentiation. Considering that HDAC4 also bound to β-catenin (Fig. 5H), we checked whether HDAC4 could form a complex with β-catenin and PLZF in SPCs. Co-IP showed no direct binding between β-catenin and PLZF (Fig. 6B), suggesting that HDAC4 might bind to β-catenin and PLZF separately. Although knockdown of *β-catenin* or *Hdac4* led to SPCs differentiation (Fig. 5I–L), it was not clear how the β-catenin-HDAC4 complex involved in this biological process, nor the role of HDAC4-PLZF complex. As a type of ubiquitous deacetylase, HDAC family members generally bind to target gene to repress gene expression through modulating its acetylation level [33], and our previous work revealed that PLZF could repress SPCs differentiation via direct binding to the promoter regions of *c-Kit* and *Stra8* [7]. Thus, dual luciferase report assay was performed to test whether HDAC4 could regulate *c-Kit* or *Stra8* expression through directly binding to their promoter regions. The *c-Kit* promoter region from − 1846 bp to − 6 bp was subcloned into pGL3 basic plasmid, and then transfected into HEK 293T cells with the recombinant HDAC4 and/or PLZF overexpression plasmids. As shown in Fig. 6C, the relative luciferase activity was remarkably declined in *pGL3-c-Kit* when co-transfected with *Plzf*, and further decreased in *Hdac4* co-transfected group. Surprisingly, simultaneous co-transfection of *Plzf* and *Hdac4* overexpression plasmids showed no further suppression of *c-Kit* activity compared to *Hdac4* transfected group, which may probably due to a more significant inhibitory effect of HDAC4 on *c-Kit* than PLZF. Similarly, the inhibition effect was also observed on *Stra8* (Fig. 6D), further confirmed the co-regulatory mechanism of HDAC4 on SPCs differentiation. Considering that HDAC4 overexpression demonstrated more efficient suppression of *c-Kit* and *Stra8* than PLZF, we hypothesized that the deacetylation level might be dependent on the gene transcription activity. Therefore, we measured acetylation levels of c-Kit and STRA8 using acetylation lysine immunoprecipitation coupled with Western blot analysis against c-Kit or STRA8. The acetylation levels of c-Kit and STRA8 in *Hdac4* knockdown group remarkably increased compared to that of control group (Fig. 6E). Thus, we postulated that HDAC4 synergically suppressed SPCs differentiation with PLZF through direct binding to differentiation associated genes (such as *c-Kit* and *Stra8*) and regulating acetylation (Fig. 6F).

Collectively, a putative regulatory pattern of E-cadherin on SPCs fate through β-catenin and HDAC4 is summarized (Fig. 6G). E-cadherin plays structural and signaling roles in SPCs. Proliferation, differentiation and apoptosis are inhibited since SPCs are attached in the niche by E-cadherin. Under the physiological condition, β-catenin is dynamically balanced among three statuses: binding to cadherins anchored at cell membrane, residing in cytoplasm and ultimately going into degradation by APC, or translocation into nucleus for transcriptional activity. In the nucleus of SPCs, β-catenin interacts with TCF/LEF, HDAC4 or STAT3 to inhibit differentiation and regulate proliferation. Based on our observations, deficiency of *E-cadherin* reduces cellular contents of β-catenin and its phosphorylation level, resulting in less β-catenin for degradation and nuclear localization. Consequently, the downstream targets associated with undifferentiation state of SPCs are disturbed. Meanwhile, the synergetic effect on inhibition of differentiation genes with HDAC4 or STAT3 is attenuated, to further promote SPCs turning to differentiation state.

## Discussion

So far, knockout assay serves as the gold standard in understanding the role of a specific gene. Unfortunately, it may not be sufficient to unmask the regulatory mechanism of E-cadherin and β-catenin in regulating SPCs fate due to their complex interactions. There have been some controversial observations reported [16, 17], and the inconsistent results regarding the role of E-cadherin in SPCs maintenance might be due to the artifacts from different knockout models, or the time duration of E-cadherin deficiency lasted in SPCs.

The complicated interaction pattern between E-cadherin and Wnt signaling pathway shadows our understanding in regulation of SPCs fate. E-cadherin is negatively regulated by Wnt signaling [34], while Wnt ligands compete with E-cadherin in binding to β-catenin. Some of Wnt’s downstream target genes negatively regulate cadherin genes [35], or further encode enzymes to destabilize membrane anchored β-catenin [36]. Cleavage of E-cadherin by proteases led to release of β-catenin from cell membrane and enhanced its transcriptional activity [37]. In reproductive organs specifically, a study showed no significant effect of *E-cadherin* knockout on SPCs homing and colonization [14]. Using an adenovirus mediated gene delivery system *E-cadherin* knockout was triggered by virus injection, following by transient transplantation of harvested SPCs. Due to the relative low efficiency (41–75%) in gene deletion, the un-infected SPCs populations seemed to be able to reconstitute germ cell pool afterwards. In our study, LoxP-Cre system was employed to enable a conditional knockout, in which the germline specific deletion of *E-cadherin* started at embryonic stage driven by *Ddx4*-Cre. Compared to the in vitro deletion system mediated by adenovirus, *Ddx4*-Cre exerts deletion as early as embryonic day 15 with a more than 95% efficacy [38]. Notably, although these mice were fertile, their fertility was slightly hampered by about 20%, and the number of PLZF^+^ population was reduced in *E-cadherin* deletion group. Overall, these observations implied that E-cadherin might affect SPCs maintenance under physiological conditions. Also, the compensation effect of CDH22-β-catenin complex on SPC development predicted by Shinohara was verified in our study, indicating a delicate interaction between cadherins and Wnt signaling pathway.

To understand the biological role of E-cadherin in regulating SPCs fate, it’s essential to focus on the two major functions of E-cadherin, the structural role and signaling role. In this study, we demonstrated that conditional disfunction of *E-cadherin* in germ cells promoted differentiation, but deletion of *E-cadherin* in SPCs through CRISPR/Cas9 not only gave rise to differentiation, but also reduced anti-apoptosis capacity. We proposed that the testicular microenvironment protected SPCs from apoptosis after *E-cadherin* deletion, and SPCs in vitro lacking of the protective function of niche were susceptible to apoptosis. However, further experimental evidence is required to verify this hypothesis.

More importantly, we revealed that loss of *E-cadherin* led to a remarkable decrease of β-catenin expression and a consequent decline of translocation to nucleus for transcription. As the pivotal molecule of cadherins and Wnt signal pathways, β-catenin is involved in the regulation of SPCs fate through transcriptional activity. HDAC4 and STAT3 were identified as two novel partners of β-catenin in this study, but the role of Wnt secreted by the niche is not determined, yet. Evidence demonstrated that Wnt5a secreted by feeder cells supports SSC self-renewal through β-catenin-independent pathway [39], and Wnt3a selectively stimulates proliferation of spermatogonia progenitors, rather than SSCs population [40]. Thus, it’s interesting to figure out the connection of secreted Wnt molecules in the niche and E-cadherin/β-catenin signaling pathway in SPCs fate in future study.

Moreover, the connection of β-catenin and SSCs fate is not fully revealed. Studies from different groups reported controversial functions of β-catenin: to promote the proliferation of PLZF^+^ undifferentiated spermatogonia [16], or to stimulate the GFRA1^+^ SPCs population to differentiate into NGN3^+^ population [17]. However, these observations are likely to be coherent. Knockout of *β-catenin* caused reduced number of PLZF^+^ and c-Kit^+^ cells in seminiferous tubules without significant difference in the number of GFRA1^+^ cells [16], indicating β-catenin mainly influenced the A_al_ and differentiating spermatogonia, due to the fact that GFRA1^+^ cells primarily distributes in A_s_ and A_pr_ [41]. Therefore, we postulated that the regulatory effect of β-catenin was more restricted to the proliferation of A_al_ and differentiating spermatogonia (c-Kit^+^) populations, rather than inducing SSCs differentiation. Meanwhile the other group claimed that β-catenin promoted differentiation of SPCs instead of affecting self-renewal, supported by the observation that deletion of *β-catenin* did not change the number of GFRA1^+^ cells, but rather affected differentiation from GFRA1^+^ to NGN3^+^ population, and activation of β-catenin led to GFRA1^+^ cells loss [17]. Collectively, these observations suggest that β-catenin only regulates the fate of A_al_ and differentiating spermatogonia. However, the ID4^+^ cells, a real SSCs enriched population [42], were not analyzed in both studies, so that it may not be sufficient to draw a solid conclusion about the interplay between β-catenin and SSCs fate. In our study, deletion of *E-cadherin* down-regulated expression of PLZF and GFRA1 in SPCs, indicating that E-cadherin affected the fate of SPCs, including A_s_, A_pr_ and A_al_ spermatogonia. We noticed that the PLZF^+^ population highly overlapped with β-catenin^+^ cells, and disturbance of either PLZF or β-catenin led to up-regulation of differentiation markers represented by c-Kit. Although no direct binding was detected between PLZF and β-catenin, they were found to bind to HDAC4 separately. Molecular assays further demonstrated that HDAC4 combined with β-catenin to suppress differentiation and promote proliferation, and synergically cooperated with PLZF to regulate acetylation of *c-kit* and *Stra8* genes in SPCs.

These observations further unveil the complicated regulation pattern of SPCs fate mediated by E-cadherin/β-catenin, but there are still many questions need to be addressed. For example, deletion of *E-cadherin* in germline through LoxP-Cre system caused the down-regulation of β-catenin and up-regulation of AXIN2 [Figs. 2M, 2N and 3A], while disturbance the expression of β-catenin, HDAC4 or STAT3 in cultured SPCs inhibited the expression of AXIN2 [Fig. 5I–N]. As the direct target gene of β-catenin/TCF4 [43], expression of *Axin2* should be positively correlated to transcriptional activity of β-catenin. However, it is worth noting that that E-cadherin could simultaneously activate multiple signaling pathways [44], which might have some unknown connection with *Axin2*. Moreover, although β-catenin is the intermediate molecule of Wnt and E-cadherin signaling pathways, the target genes activated by Wnt ligands or by *E-cadherin* loss might be different. Thus, the underlying mechanism is worthy to be explored in future study. Another question is that the connection of β-catenin and other members of cadherin family in germline is largely unknown, yet. Undifferentiated SPCs and round spermatids are likely to be distinct Wnt signaling responders [45], since E-cadherin is mainly localized in SPCs. Here we postulated that the homeostasis of β-catenin might be achieved by binding to CDH22, similar to that of E-cadherin. Loss of E-cadherin or CDH22 passively reduced the level of β-catenin, which subsequently affected its nuclear expression associated with transcriptional regulation or interaction with co-factors, leading to SPCs differentiation. These observations introduced a novel regulatory pattern of β-catenin in SPCs and may be worth looking into. Interestingly, we noticed that inhibition of E-cadherin led to up-regulated CDH22, while knockdown of *Cdh22* caused a slightly increase in E-cadherin expression (Fig. 3J, K). A compensation was speculated due to the important role of cadherins in SPC development. Notably, the binding of CDH22 and β-catenin was detected in mouse SPCs, confirming CDH22 possessed catenin binding domain in mouse SPCs, which is consistent with previous studies showing CDH22 in mouse FGSCs contained a catenin binding domain [28], but different from that in rat SSCs [27]. In mouse FGSCs, CDH22 interacts with β-catenin, JAK2 and PI3K [46], indicating that CDH22 regulates FGSCs fate via multiple signal pathways. Here, β-catenin and CDH22 are possible interactive partners as well, but the complicated network needs to be further studied.

## Conclusions

Collectively, we focused on the regulatory mechanism of E-cadherin in SPCs, demonstrating a potential regulatory pattern of SPCs maintenance mediated by E-cadherin and CDH22 through the pivotal intermediate molecule β-catenin, and revealed HDAC4 and STAT3 as the co-regulatory factors of β-catenin. We hope this study could share some novel insights into the cadherin and β-catenin-mediated SPCs fate regulation, while further research emphasizing on more detailed mechanism is acquired to enable a comprehensive understanding.

## Experimental procedures

### Animals

The CD-1 mice for experiments were supplied by Comparative Medicine Centre of Yangzhou University. The *E-cadherin* floxed mice (*Cdh1*^*L/L*^) were purchased from Jackson Lab. The protocols for breeding, mating, and genotyping of *E-cadherin* floxed mice were identical to previous study [47]. All the procedures for animal experiments were approved by the ethical committee at Nanjing Agricultural University.

### Isolation and culture of mouse SPCs

Testicular cells were extracted from 5 days postpartum mice generally following previous protocol [26]. Briefly, testes were cut into small particles and followed by collagenase IV and trypsin digestion and centrifugation, and cell pellet was resuspended and were filtered with 70-µm cell filter and subsequently sorted using mouse THY-1.2 antibody coated magnetic beads (BD, Cat.551518). Thy1.2^+^ fraction was collected and cultured on mitotically inactivated mouse embryonic fibroblast (MEF) feeder layer at 37 ℃ with 5% CO_2_. Preparation of culture medium for SPCs and making of MEF feeder cells also followed with previous protocols. SPCs could be stably maintained on MEF feeder layers for more than 30 passages.

### RNA extraction, RT-PCR and real time quantitative PCR (RT-qPCR)

For RNA extraction, tissue or cell samples were treated with TRNZol (Tiangen, DP405), and cDNA was reversed-transcripted using GoScript™ Reverse Transcription System (Promega, A5001). RT-qPCR was performed using TB Green premix Ex Taq II (Takara, RR820A) according to the instruction. Reactions were run in triplicate in three independent experiments. The results were analyzed using the 2^−△△CT^ method, and housekeeping gene *Gapdh* was used to control the variability in expression levels. The information of primers was listed in Additional file 2: Table S1.

### Immunohistochemistry (IHC) and immunofluorescence (IF)

The protocol for IHC is identical to previous study [13]. Briefly, mouse testes were harvested and fixed in 4% neutral paraformaldehyde overnight, and subsequently dehydrated and embedded in paraffin. Histological sections were dewaxed and rehydrated in ethanol series, followed by microwave antigen retrieval in 0.01 M citrate (pH = 6.0) and methanol/H_2_O_2_ treatment. After blocking with 5% goat serum, the slides were incubated with diluted primary and biotin labeled secondary antibodies, respectively. Streptavidin-HRP (Jackson Lab, 1:500) and DAB kit (Vector, sk4100) were used for visualization.

For cell IF staining, primary SPCs (within 5 passages) were carried out as described [13]. For membrane protein, 10% Neutral Formalin without Triton X-100 was used for cell fix, while for cytoplasm or nuclear protein, Carnoy’s fixative was used. Mouse IgG and rabbit IgG were used as negative control (Bioss, bs-0295PC, bs-0296PC). See antibodies information in Additional file 3: Table S2.

### Construct of recombinant plasmids and transfection

For HDAC4 and PLZF overexpression plasmid, the open reading frame (ORF) of *Hdac4* or *Plzf* were amplified and cloned into the pcDNA 3.1(+) plasmid, named as pcDNA 3.1(+)/*Hdac4* or pcDNA3.1(+)/*Plzf*. For luciferase plasmid, *c-Kit* or *Stra8* promoter gene sequences were amplified from genomic DNA using PCR, and inserted into the pGL3 basic plasmid with T4 DNA ligase (Takara, 2011A). There plasmids were nominated as pGL3 basic/*c-Kit* or pGL3 basic/*Sta8* promoter. Both of amplified sequences in the recombinant plasmids were confirmed by sequencing. Primer sequences were listed in Additional file 2: Table S1.

### RNAi and CRISPR/Cas9-mediated gene editing

For transfection and CRISPR/Cas9 assays, SPCs of 10–15 passages were used. SPCs were transfected with siRNA or plasmids using lipofectamine 3000 (Life technologies, L3000015) according to the manufacturer’s instructions. The sequences of siRNA used in this study were listed in Additional file 4: Table S3.

SPCs with targeted knockout in *Cdh1* were generated using CRISPR/Cas9 approach. SgRNAs designed using the CRISPR/Cas9 Tool (http://crispr.mit.edu) were cloned into pSpCas9(BB)-2A-Puro (PX459). The lentivirus packaging system for infection was (CSII-EF-MCS-IRES2-Venus, pCMV-VSVG-RSV-REV, pCAG-HIVgp). 293T cells were transfected with these four plasmids using Lipofectamine 3000 (Thermo Fisher, L3000008), and lentivirus in the supernatant was harvested 72 h later and concentrated with a GML-PCTM kit (Genomeditech Shanghai, GM-040801-15). We performed virus titer assay and infected SPCs following previous protocol [13]. Western blotting was used to confirm the efficiency of knockout. The sgRNA sequences are listed in Additional file 4: Table S3.

### Western blot (WB) and Co-Immunoprecipitation (Co-IP)

The protocols for WB and Co-IP are identical to previous study [13], and details are summarized below:

Protein samples separated by SDS-PAGE were electro-transferred to PVDF membrane, which were subsequently blocked in 5% skim milk at room temperature for 1 h and then incubated with primary antibodies overnight at 4 ℃. After that, the membranes were rinsed in TBST followed by incubated with goat anti-mouse IgG-HRP (Santa Cruz, sc-2005) or mouse anti-rabbit IgG-HRP (Santa Cruz, sc-2357). Finally, samples were visualized using enhanced chemiluminescence (Tanon, 180-501). The information of antibodies was listed in Additional file 3: Table S2.

All procedures were conducted at 4 °C to preserve the protein integrity. Cells were lysed in lysis buffer (Beyotime, P0013) with gentle rocking. After centrifugation, the supernatant was collected and transferred to new tubes. Thereafter, the supernatant was incubated with the diluted antibodies overnight with a gentle rotation. Protein A/G agarose beads (Santa Cruz, sc-2003) were added into the supernatant and incubated for 3 h. Afterwards, beads were precipitated by centrifugation and washed five times with the cold lysis buffer. Finally, the pellet was resuspended in 1 × SDS loading buffer followed by western blot analysis.

For analysis of samples from mouse testes, three 90-day *E-cadherin*^L/L^ mice and three 90-day *E-cadherin*^L/L^;*Ddx4-Cre*^+^ mice were sacrificed to collect testes, and each testis was separately treated for Western blot analysis. For cell samples, SPCs of 3–5 wells in 24-well plates were harvested (around 10^6) for analysis, and all experiments repeated at least three times.

### Dual luciferase reporter assay

The dual luciferase reporter assay was performed in triplicate based on a previous protocol [48]. Briefly, 0.25 μg of pGL3 basic/*Kit* promoter, 0.25 μg of empty pcDNA 3.1(+), pcDNA 3.1(+)/*Hdac4* or pcDNA 3.1(+)/*Plzf* and 3 ng of an internal control *Renilla* luciferase assay vector pRL-CMV were transfected into HEK 293T cells. Cells were pre-seeded in 24-well plates at a concentration of 4 × 10^4^ per well. Twenty-four hours post-transfection, luciferase activity was measured with a dual luciferase kit (Promega, E1910) by the Glomax® 20/20 luminometer (Promega, E5311). Three wells were prepared for each experiment, and the experiments were repeated for three times. The results were calculated by normalizing the luciferase fluorescence value to that of renilla fluorescence.

### Statistical analysis

For cell counting, sections or immunofluorescent visual fields were selected randomly. All the spermatogonia in 200× magnification view of microscope were counted and statistically analyzed, and the ratio of SPCs number in knockdown group/control group was defined as relative SPCs number. For IHC, the positive cells in twelve seminiferous tubules of slides were counted under microscope. The seminiferous tubules were randomly selected in discontinuous slides from three mice. Values plotted were expressed as mean ± standard deviation (SD). Statistical analysis was performed using Graphpad prism7 and Student’s *t*-test, *p < 0.05; **p < 0.01.

## Supplementary Information

**Additional file 1: Figure S1.** Determination of E-cadherin, β-catenin, Axin2 and ZO-2 in SPCs. The expression of PLZF (A), E-cadherin (B), β-catenin (C), AXIN2 (D) and ZO-2 (E) was detected in purified SPCs using IF staining. Scale bar = 20 μm.

**Additional file 2: Table S1.** Information of primers used in this study.

**Additional file 3: Table S2.** Antibody information in this study.

**Additional file 4: Table S3.** The sequences of siRNA and sgRNA used in this study.

## Data Availability

The data that supports the findings of this study are available in the method part and supplemental materials.

## Abbreviations

CDH: Cadherin
Co-IP: Co-Immunoprecipitation
LEF1: Lymphoid enhancer-binding factor 1
MACS: Magnetic activated cell sorting
FGSCs: Female germline stem cells
IF: Immunofluorescence
PLZF: Promyelocytic leukemia zinc finger
RT-qPCR: Real time quantitative PCR
RT-PCR: Reverse Transcription-Polymerase Chain Reaction
RNAi: RNA interference
SSCs: Spermatogonial stem cells
TCF: T-cell factor
WB: Western blot

## References

[1] Hobbs RM, Fagoonee S, Papa A (2012). Functional antagonism between Sall4 and Plzf define germline progenitors. Cell Stem Cell.

[2] Costoya JA, Hobbs RM, Maria B (2004). Essential role of PLZF in maintenance of spermatogonial stem cells. Nat Genet.

[3] Hermann BP, Sukhwani M, Lin CC (2007). Characterization, cryopreservation, and ablation of spermatogonial stem cells in adult rhesus macaques. Stem Cells.

[4] Buaas FW, Kirsh AL, Sharma M (2004). Plzf is required in adult male germ cells for stem cell self-renewal. Nat Genet.

[5] Filipponi D, Hobbs RM, Ottolenghi S (2007). Repression of kit expression by Plzf in germ cells. Mol Cell Biol.

[6] Hobbs RM, Seandel M, Falciatori I (2010). Plzf regulates germline progenitor self-renewal by opposing mTORC1. Cell.

[7] Song W, Shi X, Xia Q (2020). PLZF suppresses differentiation of mouse spermatogonial progenitor cells via binding of differentiation associated genes. J Cell Physiol.

[8] Yan HH, Mruk DD, Lee WM (2008). Cross-talk between tight and anchoring junctions-lesson from the testis. Adv Exp Med Biol.

[9] Nelson WJ, Nusse R (2004). Convergence of Wnt, beta-catenin, and cadherin pathways. Science.

[10] Tokuda M, Kadokawa Y, Kurahashi H (2007). CDH1 is a specific marker for undifferentiated spermatogonia in mouse testes. Biol Reprod.

[11] Carlomagno G, van Bragt MP, Korver CM (2010). BMP4-induced differentiation of a rat spermatogonial stem cell line causes changes in its cell adhesion properties. Biol Reprod.

[12] Nakagawa T, Sharma M, Nabeshima Y (2010). Functional hierarchy and reversibility within the murine spermatogenic stem cell compartment. Science.

[13] Wang J, Li J, Xu W (2019). Androgen promotes differentiation of PLZF+ spermatogonia pool via indirect regulatory pattern. Cell Commun Signal.

[14] Kanatsu-Shinohara M, Takehashi M, Takashima S (2008). Homing of mouse spermatogonial stem cells to germline niche depends on β1 integrin. Cell Stem Cell.

[15] Piprek RP, Kolasa M, Podkowa D (2019). Tissue-specific knockout of E-cadherin (Cdh1) in developing mouse gonads causes germ cells loss. Reproduction.

[16] Takase HM, Nusse R (2016). Paracrine Wnt/β-catenin signaling mediates proliferation of undifferentiated spermatogonia in the adult mouse testis. Proc Natl Acad Sci USA.

[17] Tokue M, Ikami K, Mizuno S (2017). SHISA6 confers resistance to differentiation-promoting Wnt/β-catenin signaling in mouse spermatogenic stem cells. Stem Cell Rep.

[18] Chassot AA, Le Rolle M, Jourden M (2017). Constitutive WNT/CTNNB1 activation triggers spermatogonial stem cell proliferation and germ cell depletion. Dev Biol.

[19] Kumar M, Atkins J, Cairns M (2016). Germ cell-specific sustained activation of Wnt signalling perturbs spermatogenesis in aged mice, possibly through non-coding RNAs. Oncotarget.

[20] Boyer A, Zhang X, Levasseur A (2021). Constitutive activation of CTNNB1 results in a loss of spermatogonial stem cell activity in mice. PLoS ONE.

[21] Sineva GS, Pospelov VA (2014). β-Catenin in pluripotency: adhering to self-renewal or Wnting to differentiate?. Int Rev Cell Mol Biol.

[22] Harris TJC, Peifer M (2005). Decisions, decisions: β-catenin chooses between adhesion and transcription. Trends Cell Biol.

[23] Valenta T, Hausmann G, Basler K (2012). The many faces and functions of β-catenin. EMBO J.

[24] Kofman AE, Huszar JM, Payne CJ (2013). Transcriptional analysis of histone deacetylase family members reveal similarities between differentiating and aging spermatogonial stem cells. Stem Cell Rev Rep.

[25] Chauchereau A, Mathieu M, Saintignon JD (2004). HDAC4 mediates transcriptional repression by the acute promyelocytic leukaemia-associated protein PLZF. Oncogene.

[26] Wei R, Zhang X, Cai Y (2020). Busulfan suppresses autophagy in mouse spermatogonial progenitor cells via mTOR of AKT and p53 signaling pathways. Stem Cell Rev Rep.

[27] Wu J, Zhang Y, Tian GG (2008). Short-type PB-cadherin promotes self-renewal of spermatogonial stem cells via multiple signaling pathways. Cell Signal.

[28] Zhang X, Yang Y, Xia Q (2018). Cadherin 22 participates in the self-renewal of mouse female germ line stem cells via interaction with JAK2 and β-catenin. Cell Mol Life Sci.

[29] Sharma A, Chen Q, Nguyen T (2012). T cell factor-1 and β-catenin control the development of memory-like CD8 thymocytes. J Immunol.

[30] Iaconelli J, Xuan L, Karmacharya R (2019). HDAC6 modulates signaling pathways relevant to synaptic biology and neuronal differentiation in human stem-cell-derived neurons. Int J Mol Sci.

[31] Godmann M, May E, Kimmins S (2010). Epigenetic mechanisms regulate stem cell expressed genes Pou5f1 and Gfra1 in a male germ cell line. PLoS ONE.

[32] GarcíadeHerreros A, Duñach M (2019). Intracellular signals activated by canonical Wnt ligands independent of GSK3 inhibition and β-catenin stabilization. Cells.

[33] Wang AH, Bertos NR, Vezmar M (1999). HDAC4, a human histone deacetylase related to yeast HDA1, is a transcriptional corepressor. Mol Cell Biol.

[34] Jamora C, DasGupta R, Kocieniewski P (2003). Links between signal transduction, transcription and adhesion in epithelial bud development. Nature.

[35] Howe LR, Watanabe O, Leonard J (2003). Twist is up-regulated in response to Wnt1 and inhibits mouse mammary cell differentiation. Cancer Res.

[36] Malliri A, Rygiel TP, van der Kammen RA (2006). The rac activator Tiam1 is a Wnt-responsive gene that modifies intestinal tumor development. J Biol Chem.

[37] Maretzky T, Reiss K, Ludwig A (2005). ADAM10 mediates E-cadherin shedding and regulates epithelial cell-cell adhesion, migration, and beta-catenin translocation. Proc Natl Acad Sci USA.

[38] Gallardo T, Shirley L, John GB (2007). Generation of a germ cell-specific mouse transgenic Cre line, Vasa-Cre. Genesis.

[39] Yeh JR, Zhang X, Nagano MC (2011). Wnt5a is a cell-extrinsic factor that supports self-renewal of mouse spermatogonial stem cells. J Cell Sci.

[40] Yeh JR, Zhang X, Nagano MC (2012). Indirect effects of Wnt3a/β-catenin signalling support mouse spermatogonial stem cells in vitro. PLoS ONE.

[41] Masaki K, Sakai M, Kuroki S (2018). FGF2 has distinct molecular functions from GDNF in the mouse germline niche. Stem Cell Rep.

[42] Helsel AR, Yang QE, Oatley MJ (2017). ID4 levels dictate the stem cell state in mouse spermatogonia. Development.

[43] Yu Y, Wu J, Wang Y (2012). Kindlin 2 forms a transcriptional complex with β-catenin and TCF4 to enhance Wnt signalling. EMBO Rep.

[44] Onder TT, Gupta PB, Mani SA (2008). Loss of E-cadherin promotes metastasis via multiple downstream transcriptional pathways. Cancer Res.

[45] Kerr GE, Young JC, Horvay K (2014). Regulated Wnt/beta-catenin signaling sustains adult spermatogenesis in mice. Biol Reprod.

[46] Zhang X, Wei R, Sun Y (2019). AKT3 is a pivotal molecule of cadherin-22 and GDNF family receptor-α1 signal pathways regulating self-renewal in female germline stem cells. Stem Cells.

[47] Olson A, Le V, Aldahl J (2019). The comprehensive role of E-cadherin in maintaining prostatic epithelial integrity during oncogenic transformation and tumor progression. PLoS Genet.

[48] Qian Y, Jung Y-S, Chen X (2012). Differentiated embryo-chondrocyte expressed gene 1 regulates p53-dependent cell survival versus cell death through macrophage inhibitory cytokine-1. Proc Natl Acad Sci USA.

