# Supplementary figures and images for: E-cadherin maintains the undifferentiated state of mouse spermatogonial progenitor cells via β-catenin
Source: Cell Biosci. 2022 Sep 1;12:141. doi: 10.1186/s13578-022-00880-w (PMC9434974; doi:10.1186/s13578-022-00880-w)

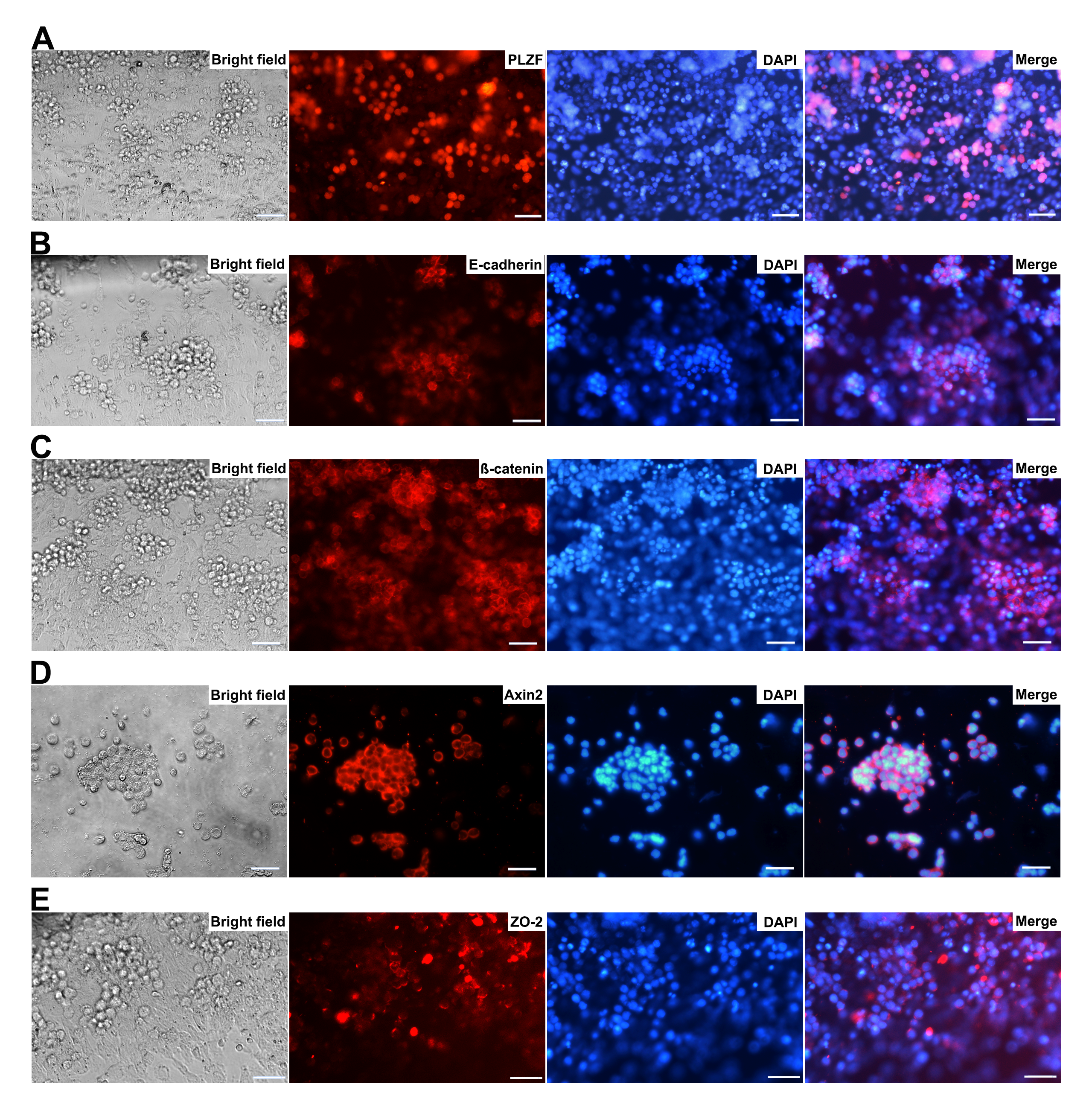

Supplement: Supplementary file 1 — Additional file 1: Figure S1. Determination of E-cadherin, β-catenin, Axin2 and ZO-2 in SPCs. The expression of PLZF (A), E-cadherin (B), β-catenin (C), AXIN2 (D) and ZO-2 (E) was detected in purified SPCs using IF staining. Scale bar = 20 μm. [file 13578_2022_880_MOESM1_ESM.tif]
