# Supplementary material for: E-cadherin maintains the undifferentiated state of mouse spermatogonial progenitor cells via β-catenin
Source: Cell Biosci. 2022 Sep 1;12:141. doi: 10.1186/s13578-022-00880-w (PMC9434974; doi:10.1186/s13578-022-00880-w)
Supplement: Supplementary file 2 — Additional file 2: Table S1. Information of primers used in this study. [file 13578_2022_880_MOESM2_ESM.docx]

**Table S1. Information of primers used in this study.**

| RT-PCR primer sequences | | | | | | |  |
| --- | --- | --- | --- | --- | --- | --- | --- |
| Gene | Accession number | | Product  Size(bp) | Primer Sequence(5’-3’) | | Region Amplified |  |
| *Gfrα1* | NM_010279.3 | | 191bp | F: ACTCCTGGATTTGCTGATGTCGG | | 480-670 |  |
|  |  | |  | R: CGCTGCGGCACTCATCCTT | |  |  |
| *Plzf* | NM_001033324.3 | | 177bp | F: CACACTCAAGAGCCACAAGC | | 1920-2096 |  |
|  |  | |  | R: ATCATGGCCGAGTAGTCTCG | |  |  |
| *Cdh1* | NM_009864.3 | | 217bp | F: ACCGATTCAAGAAGCTGGC | | 2751-2966 |  |
|  |  | |  | R: ACCATCCTAACACAGACAGTCC | |  |  |
| *Id4* | NM_031166.3 | | 151bp | F: TGCAGTGCGATATGAACGAC | | 262-412 |  |
|  |  | |  | R: AAAGCAGGGTGAGTCTCCAG | |  |  |
| *Gapdh* | NM_008084.3 | | 133bp | F: CCTGGAGAAACCTGCCAAGTATG | | 978-1087 |  |
|  |  | |  | R: AGAGTGGGAGTTGCTGTTGAAGTC | |  |  |
| *Hdac1* | [NM_008228.2](https://www.ncbi.nlm.nih.gov/nuccore/NM_008228.2) | | 167bp | F：TCCAACATGACCAACCAGAA | | 1059-1225 |  |
|  |  | |  | R：TTGTCAGGGTCCTCCTCATC | |  |  |
| *Hdac2* | [NM_008229.2](https://www.ncbi.nlm.nih.gov/nuccore/NM_008229.2) | | 245bp | F：TGGAGGAGGCTACACAATCC | | 1108-1352 |  |
|  |  | |  | R：TTTGAACACCAGGTCCATGT | |  |  |
| *Hdac3* | [NM_010411.2](https://www.ncbi.nlm.nih.gov/nuccore/NM_010411.2) | | 238bp | F：CCCCTTTCCCTCAAACTCTC | | 1514-1751 |  |
|  |  | |  | R：TTGCATGGAAGCAAGAACTG | |  |  |
| *Hdac4* | [NM_053449.1](https://www.ncbi.nlm.nih.gov/nuccore/NM_053449.1) | | 204bp | F：TATGGAATGAGGTGCACTCGTCTGG | | 2559-2762 |  |
|  |  | |  | R：GCCTCTGCTGGAGAAGTTTGGC | |  |  |
| *Hdac5* | [NM_001077696.1](https://www.ncbi.nlm.nih.gov/nuccore/NM_001077696.1) | | 199bp | F：GGAGACAGAAGAGGAGCTGACG | | 1994-2192 |  |
|  |  | |  | R：AGGGCCTTCATCAGGACCACTCTC | |  |  |
| *Hdac6* | [NM_001130416.1](https://www.ncbi.nlm.nih.gov/nuccore/NM_001130416.1) | | 152bp | F：TGAGTCACTGCAACCTCTGG | | 1736-1887 |  |
|  |  | |  | R：GTGGCAGGTAAGGAGCTCAG | |  |  |
| *Hdac7* | [NM_001204275.1](https://www.ncbi.nlm.nih.gov/nuccore/NM_001204275.1) | | 201bp | F：GTAGCTGCTCTTCTGGGCAACAAGG | | 3016-3216 |  |
|  |  | |  | R：CACGGCTTCCACTTCTGCATCAGC | |  |  |
| *Hdac8* | [NM_001313742.1](https://www.ncbi.nlm.nih.gov/nuccore/NM_001313742.1) | | 215bp | F：GCCATCAACTGGTCTGGAGGGTG | | 494-708 |  |
|  |  | |  | R：GGGGAGAACTTGTGCAGGGACAC | |  |  |
| *Hdac9* | [NM_024124.3](https://www.ncbi.nlm.nih.gov/nuccore/NM_024124.3) | | 190bp | F：CGCGTAGGCAGACATGTAGA | | 3261-3450 |  |
|  |  | |  | R：ACCTGTCCAACAAGGCAAAC | |  |  |
| *Tcf3* | NM_001079822.2 | | 268bp | F：GGAGCCGGGGCAACCAGTG | | 151-418 |  |
|  |  | |  | R： CATCCTGGGGCCTTCTCACTTC | |  |  |
| *Tcf4* | NM_001142918.2 | | 201bp | F： GCCAAGAGGCAAGATGGAG | | 791-991 |  |
|  |  | |  | R：ACGAGCATCCTTGAGGGTTT | |  |  |
| *Tcf7* | NM_001313981.1 | | 115bp | F：AGCTTTCTCCACTCTACGAACA | | 205-319 |  |
|  |  | |  | R：AATCCAGAGAGATCGGGGGTC | |  |  |
| *Lef1* | NM_001276402.1 | | 140bp | F：CTGCGGGCTGGAACATTT | | 342-481 |  |
|  |  | |  | R：CGGAGGAGGAGGGGAGAA | |  |  |
| qRT-PCR primer sequences | | | | | | | |
| Gene | | Forward | | | Reverse | | |
| *Cdh1* | | AGCCATTGCCAAGTACATCC | | | TCTGGCCTGTTGTCATTCTG | | |
| *Axin2* | | GGTTCCGGCTATGTCTTTGC | | | CAGTGCGTCGCTGGATAACTC | | |
| *β-catenin* | | TGACCAGTTCCCTCTTCAGG | | | ATGCTCCATCATAGGGTCCA | | |
| *Plzf* | | GCAGCTATATTTGCAGTGAG | | | TCTTGAGTGTGCTCTCATCC | | |
| *Kit* | | TGGAGTTTCCCAGAAACA | | | AAATGGGCACTTGGTTTGA | | |
| *Gapdh* | | AACTTTGGCATTGTGGAAGG | | | ACACATTGGGGGTAGGAACA | | |
|  | | | | | | | |
| open reading frame (ORF) and promoter primer sequences | | | | | | | |
| Gene | | Forward | | | Reverse | | |
| *Hdac4* ORF | | CGGAATTCAACTTAAGGCACTGACGCTG | | | TTGCGGCCGCGCAGGACGCAGGAGTGAT | | |
| *Plzf* ORF | | CGGAATTCCAGAGAAGGAAAGAGAGCCC | | | TTGCGGCCGCTTGTCTGGTTCTAGCTCGC | | |
| *Stra8* promoter | | CGACGCGTGCTACTCAGCAGACTCAT | | | TTCTCGAGTGAGGAAGGCAGACAGAGC | | |
| *c-Kit* promoter | | GCGACGCGTCCACCTATCTACCTACCT | | | TTGCTCGAGTGGCTGCGCTAGACTCT | | |
