# Supplementary material for: E-cadherin maintains the undifferentiated state of mouse spermatogonial progenitor cells via β-catenin
Source: Cell Biosci. 2022 Sep 1;12:141. doi: 10.1186/s13578-022-00880-w (PMC9434974; doi:10.1186/s13578-022-00880-w)
Supplement: Supplementary file 3 — Additional file 3: Table S2. Antibody information in this study. [file 13578_2022_880_MOESM3_ESM.docx]

**Table S2. Antibody information in this study**

| **Antibody** | **Company** | **Catalog** |
| --- | --- | --- |
| Polyclonal mouse anti-rabbit PLZF | Santa Cruz | sc-22839 |
| Monoclonal mouse anti-mouse E-cadherin | Abcam | ab76055 |
| Polyclonal mouse anti-rabbit ITGA6 (H-87) | Santa Cruz | sc-10730 |
| Polyclonal mouse anti-rabbit GFRA1 | Affinity | DF7309 |
| Monoclonal mouse anti-mouse PLZF | Santa Cruz | sc-28319 |
| Monoclonal mouse anti-rabbit β-catenin | Cell Signaling Technology | #8480 |
| Monoclonal mouse anti-rabbit HDAC4 | Cell Signaling Technology | #7628 |
| Polyclonal mouse anti-rabbit STAT3 | Cell Signaling Technology | #9132 |
| Polyclonal mouse anti-rabbit CDH22 | Affinity | AF3529 |
| Monoclonal mouse anti-rabbit c-Kit | Cell Signaling Technology | #3074 |
| Monoclonal mouse anti-mouse Gsk3-β | Santa Cruz | sc-7291 |
| Monoclonal mouse anti-Rabbit CyclinD1 | ABclonal | A19038 |
| Polyclonal mouse anti-rabbit BCL-2 | Beyotime | AB112 |
| Monoclonal mouse anti-rabbit BAX | HUABIO | ET1603-34 |
| Monoclonal mouse anti-rabbit PCNA | HUABIO | ET1605-38 |
| Polyclonal mouse anti-rabbit STRA8 | Abcam | ab49602 |
| Monoclonal mouse anti-rabbit TCF7L2 | Beyotime | AF2089 |
| Polyclonal mouse anti-rabbit LEF1 | Proteintech | 14972-1-AP |
| Polyclonal mouse anti-rabbit TCF7L1 | Proteintech | 14519-1-AP |
| Monoclonal mouse anti-mouse β-tubulin | Anbo | P07437 |
| Monoclonal mouse anti-rabbit Phospho-β-Catenin(Ser675) | Cell Signaling Technology | #4176 |
| Polyclonal mouse anti-rabbit Phospho-β-Catenin(S33/S37/T41) | Cell Signaling Technology | #9561 |
| Polyclonal mouse anti-rabbit SOHLH2 | Bioss | bs-12279R |
| Polyclonal mouse anti-rabbit AXIN2 | Absin | abs116112 |
| Monoclonal mouse anti-mouse ZO-2 | Santa Cruz | sc-515115 |
| Pan Acetyl-Lysine Rabbit pAb | Abclonal | A2391 |
| Monoclonal mouse anti-mouse Histone H3 | Beyotime | AF0009 |
| Alexa Flour532 goat anti-rabbit IgG (H+L) | Invitrogen | A-11009 |
| Alexa Flour555 goat anti-mouse IgG (H+L) | Invitrogen | A-21422 |
| goat anti-mouse IgG-FITC | ZSGB-Bio | ZF-0312 |
| goat anti-rabbit IgG-FITC | Beyotime | A0562 |
| HRP-linked goat anti-rabbit IgG (H&L) | Santa Cruz | sc2004 |
| HRP-linked goat anti-mouse IgG (H&L) | Santa Cruz | sc2005 |
| Biotin labelled goat anti-mouse IgG | Vector | BA-9200 |
| Biotin labelled goat anti-rabbit IgG | Vector | BA-1000 |
