# Supplementary material for: E-cadherin maintains the undifferentiated state of mouse spermatogonial progenitor cells via β-catenin
Source: Cell Biosci. 2022 Sep 1;12:141. doi: 10.1186/s13578-022-00880-w (PMC9434974; doi:10.1186/s13578-022-00880-w)
Supplement: Supplementary file 4 — Additional file 4: Table S3. The sequences of siRNA and sgRNA used in this study. [file 13578_2022_880_MOESM4_ESM.docx]

**Table S3. The sequences of siRNA and sgRNA used in this study**

| **siRNA** | **Sense（5'-3'）** |
| --- | --- |
| *E-cadherin* #1 | UGAGAAGUCCCCCAGCCAG |
| *E-cadherin* #2 | CUGGCUGGGGGACUUCUCA |
| *Cdh22* #1 | GGACAUCAACGACAGUGAATT |
| *Cdh22* #2 | UUCACUGUCGUUGAUGUCCTT |
| *Hdac4* #1 | CACAGUUGCAUGAACAUAU |
| *Hdac4* #2 | AUAUGUUCAUGCAACUGUG |
| *β-catenin* #1 | ACAUAAUGAGGACCUACACTT |
| *β-catenin* #2 | GUGUAGGUCCUCAUUAUGUTT |
| *Lef1* #1 | GGCCAAAUACUAUGAACUATT |
| *Lef1* #2 | UAGUUCAUAGUAUUUGGCCTT |
| *Tcf3* #1 | GAGAAGAAACCUCACGUGATT |
| *Tcf3* #2 | UCACGUGAGGUUUCUUCUCTT |
| *Stat3* #1 | CCUCCAGGACGACUUUGAU |
| *Stat3* #2 | AUCAAAGUCGUCCUGGAGG |
| Scrambled #1 | UUCUCCGAACGUGUCACGUTT |
| Scrambled #2 | ACGUGACACGUUCGGAGAATT |
| **sgRNA** |  |
| Screen #1 | CGCCAAGTCCAGAGCCATTTCCATC |
| Screen #2 | CACTGGAAGAGACACAATATGCACG |
| *Cdh1-sgRNA #1* | CACCGGAGCTGACAAACCCCCCGT |
| *Cdh1-sgRNA #2* | AAACACGGGGGGTTTGTCAGCTCC |
